# Subchronic Treatment of Donepezil Rescues Impaired Social, Hyperactive, and Stereotypic Behavior in Valproic Acid-Induced Animal Model of Autism

**DOI:** 10.1371/journal.pone.0104927

**Published:** 2014-08-18

**Authors:** Ji-Woon Kim, Hana Seung, Kyung Ja Kwon, Mee Jung Ko, Eun Joo Lee, Hyun Ah Oh, Chang Soon Choi, Ki Chan Kim, Edson Luck Gonzales, Jueng Soo You, Dong-Hee Choi, Jongmin Lee, Seol-Heui Han, Sung Min Yang, Jae Hoon Cheong, Chan Young Shin, Geon Ho Bahn

**Affiliations:** 1 Department of Neuroscience, School of Medicine, Konkuk University, Seoul, Korea; 2 Center for Neuroscience Research, Institute of Biomedical Science and Technology, Konkuk University, Seoul, Korea; 3 Department of Pharmacology, College of Pharmacy, Sahmyook University, Seoul, Korea; 4 Department of Neuropsychiatry, School of Medicine, Kyung Hee University, Seoul, Korea; Weizmann Institute of Science, Israel

## Abstract

Autism spectrum disorder (ASD) is a group of pervasive developmental disorders with core symptoms such as sociability deficit, language impairment, and repetitive/restricted behaviors. Although worldwide prevalence of ASD has been increased continuously, therapeutic agents to ameliorate the core symptoms especially social deficits, are very limited. In this study, we investigated therapeutic potential of donepezil for ASD using valproic acid-induced autistic animal model (VPA animal model). We found that prenatal exposure of valproic acid (VPA) induced dysregulation of cholinergic neuronal development, most notably the up-regulation of acetylcholinesterase (AChE) in the prefrontal cortex of affected rat and mouse offspring. Similarly, differentiating cortical neural progenitor cell in culture treated with VPA showed increased expression of AChE *in vitro*. Chromatin precipitation experiments revealed that acetylation of histone H3 bound to AChE promoter region was increased by VPA. In addition, other histone deacetyalse inhibitors (HDACIs) such as trichostatin A and sodium butyrate also increased the expression of AChE in differentiating neural progenitor cells suggesting the essential role of HDACIs in the regulation of AChE expression. For behavioral analysis, we injected PBS or donepezil (0.3 mg/kg) intraperitoneally to control and VPA mice once daily from postnatal day 14 all throughout the experiment. Subchronic treatment of donepezil improved sociability and prevented repetitive behavior and hyperactivity of VPA-treated mice offspring. Taken together, these results provide evidence that dysregulation of ACh system represented by the up-regulation of AChE may serve as an effective pharmacological therapeutic target against autistic behaviors in VPA animal model of ASD, which should be subjected for further investigation to verify the clinical relevance.

## Introduction

Autism spectrum disorder (ASD) is characterized by three core behavioral deficits which include symptoms such as impaired sociability, language problems, and restricted or repetitive behaviors. Although the exact mechanism and etiology for ASD is not known, a myriad of genetic and environmental risk factors of ASD has been studied in the last few decades (reviewed in [1], [2]). In spite of these endeavors to find out the etiological factors and therapeutic targets for ASD, recently approved drugs (namely risperidone and aripiprazole) have shown limited effect and not for all core symptoms, especially social defects [3].

Pipeline drugs under clinical trials for ASD are mainly focused on modulation of neurotransmitters or their related receptors. However, a drug to modulate the cholinergic system is not yet on the prime list as a candidate for therapeutics of ASD [4], [5]. Nevertheless, acetylcholine (ACh) is implicated in various neurological processes such as plasticity, cognition, memory, release of other neurotransmitters and so on, especially in the central nervous system [6], [7], [8], [9], [10]. These implications suggest sufficient motivation to investigate the possible role of ACh in ASD. Acetylcholine is synthesized with acetyl CoA and choline by the action of choline acetyl transferase (ChAT) and is degraded by acetylcholinesterase (AChE). The dysregulation of these enzymes and cholinergic receptors (both muscarinic and nicotinic acetylcholine receptors) cause many neurological disorders such as alzheimer's disease, Parkinson's disease, schizophrenia, and recently expanded to ASD [11], [12], [13], [14].

In many studies, dysregulation of cholinergic system commonly observed in the brain of ASD patient. Recently, genetic mutations in *CHRNA7* (encoding α7-nicotinic acetylcholine receptor subunit) and *CHRM3* (encoding M3 muscarinic receptor) were found in autistic patients [15], [16], [17]. In post-mortem studies with ASD patients' brain, M1 muscarinic receptor and several nicotinic receptors subunits (α3, α4, β2) were reduced but α7 subunit of nicotinic receptor was up-regulated [18], [19], [20], [21]. Along with these results, decreased choline peak was observed in the gray matter and temporal lobe of ASD patients using proton magnetic resonance spectroscopy, although this does not necessarily correspond to free choline level [22], [23]. All of these studies implicate that ASD patients may have dysregulated cholinergic system in the brain. To further strengthen the above implications, small scale clinical trials have been performed using acetylcholinesterase inhibitors (AChEIs) such as donepezil, rivastigmine, and galantamine to ASD patients [24], [25], [26], [27]. These drugs have shown to produce varying degrees of efficacy in language skill, communication, and hyperactivity. These results therefore suggest that AChEIs could be potential therapeutic entities that represent another etiological pathway for ASD. However, detailed pharmacological and neurobiological studies to investigate the proof of concept of cholinergic dysregulation in ASD are far too scarce.

In this study, we used valproic acid (VPA)-induced animal model of ASD (VPA animal) to study the dysregulation of cholinergic system and its role in the treatment of autistic behaviors. VPA is an anticonvulsant drug and mood stabilizer in clinical use but prenatal exposure to VPA has been linked to ASD along with its teratogenic effect both in humans and studied animals [28], [29]. In many studies including ours, rodents such as rats and mice prenatally exposed to VPA showed autistic behaviors; i.e. decreased sociability, increased repetitive behavior, hyperactivity, increased epileptic potential to electric shock and so on [29], [30], [31]. Thus these animals were utilized as plausible animal models for ASD more often. In this study, we found that the VPA animal models have increased AChE level in both rats and mice. From this aberrant expression level of enzymes, we hypothesized that upregulation of ACh level by inhibiting overt AChE activity with donepezil could rescue the autistic behaviors in this model. In our results, subchronic treatment of donepezil ameliorated social behavioral deficits, repetitive behavior and hyperactivity. We hereby suggest that modulating cholinergic system should be given further attention as a potential pharmacological therapeutic target against ASD.

## Materials and Methods

### Materials

Dulbecco's modified Eagle's medium/F12 (DMEM/F12) was obtained from Gibco BRL (Grand Island, NY) and B-27 supplement was purchased from Invitrogen (Carlsbad, CA). Antibodies against AChE (NBP1-51274) was from Novus biological (CO, USA), choline acetyltransferase (ChAT, MAB5350) was from Chemicon (CO, USA), histone H3 (#9715) was from Cell signaling (MA, USA), and acetyl-histone H3 was from Millipore (MA, USA). Other reagents including sodium valproate (P4543), trichostatin A(T8552), sodium butyrate(B5887), and donepezil hydrochloride monohydrate (D6821) were purchased from Sigma-Aldrich (St. Louis, MO).

### Methods

#### Cell cultures

Rat primary NPCs were isolated from cerebral cortex of embryonic day 14(E14) SD rat as described previously [32]. For differentiation, NPCs were sub-cultured into poly-L-ornithine (0.1 mg/ml) pre-coated multi-well plates (1×10^6^/ml) using B27 supplement containing DMEM/F12 medium without growth factors. The cultures were kept in a humidified atmosphere (5% CO_2_) at 37°C. HDACIs such as sodium valproate (0.5 mM), sodium butyrate (0.1 mM) and trichostatin A (0.2 µM) were treated 3 h after sub-culture. When appropriate, cell viability was determined by MTT assay. For cell culture and further progenitor cell differentiation experiments, timed pregnant female rats were obtained from OrientBio (Gyeonggi-do, Korea). Animal treatments including anesthesia, euthanasia and administration were carried out in accordance with the Principle of Laboratory Animal Care (NIH publication No. 85-23, revised 1985) and were approved by Animal Care and Use Committee of Konkuk University, Korea (KU13156).

#### Western blot analysis

Cells were washed twice with PBS and lysed with 2x SDS-PAGE sample buffer (120 mM Tris-HCl (pH 6.8), 20% glycerol, 4% SDS, 28.8 mM 2-mercaptoethanol, and 0.01% bromophenol blue). Brain tissues were homogenized using RIPA buffer (150 mM sodium chloride, 1% Triton X-100, 0.5% sodium deoxycholate, 0.1%SDS, 50 mM Tris, pH 8.0), and the resulting lysates were diluted with 2X SDS-PAGE sample buffer. Same amounts of protein (20 µg) determined by BCA protein assay was separated by 10% SDS-PAGE and transferred to nitrocellulose membranes. The membranes were blocked with 1% skim milk in TBST containing 0.2% tween-20 for 1 hr. The membranes were incubated with primary antibody overnight at 4°C and with peroxidase-conjugated secondary antibody (Santa Cruz, CA) for 2 hrs at room temperature. Specific bands were detected using the ECL system (Amersham, Buckinghamshire, UK) and exposed to Bio-Rad electrophoresis image analyzer (Bio-Rad, Hemel Hampstead, UK). β-actin was used as loading control and Western blot band intensity was normalized with β-actin immunoreactivity.

#### RT-PCR

Total RNA was extracted from NPCs using Trizol reagent and 1 µg of total RNA was reverse transcribed using a RevertAidTM Reverse transcriptase kit (K1622, Fermentas, Glen Burnie, Maryland, USA). Then 3 µl of the cDNA was used for a PCR amplification that consisted of 32 cycles (94°C, 1 min; 60°C, 30 sec; 72°C, 35 sec and continued by a final extension step at 72°C for 10 min) with the oligonucleotide primers for *Ache* [33], *Chat* [34]. For *Gapdh* (accession number: M17701) amplification, 25 cycles of PCR reaction was used.

for *Ache*,

forward primer: 5′-TTC TCC CAC ACC TGT CCT CAT C-3′

reverse primer: 5′-TTC ATA GAT ACC AAC ACG GTT CCC -3′

for *Chat*,

forward primer: 5′- CAA CCA TCT TCT GGC ACT GA-3′

reverse primer: 5′- TAG CAG GCT CCA TAG CCA TT-3′

for *Gapdh*,

forward primer: 5′- AAT GCA TCC TGC ACC ACC AA-3′

reverse primer: 5′- GAT GGC ATG GAC TGT GGT CA-3′

The amplified PCR products were analyzed on EtBr contained agarose gel and visualized.

#### Chromatin immunoprecipitation

Chromatin immunoprecipitation(ChIP) was performed according to the reported method [35] with minor modifications. For in vitro ChIP, 43 µl of 37% formaldehyde was added to 1.6 ml of overlaying culture medium of neural progenitor cell and incubated for 15 min at room temperature. After incubation, 225 µl of 1 M glycine was added and incubated for 5 min. Cells were scraped and collected by centrifugation (2,000 g for 5 min at 4°C), then washed twice with cold PBS. For in vivo tissue ChIP, prefrontal cortices of control or VPA-exposed rat offspring at week 4 were homogenized with PBS. And 43 µl of 37% formaldehyde was added to 1.6 ml of homogenates. After 15 min incubation at room temperature, 225 ul of 1 M glycine was added and incubated for 5 min. The homogenates were centrifuged (2,000 g for 5 min at 4°C), then washed twice with cold PBS. Collected cells and homogenates were lysed with IP buffer (150 mM sodium chloride, 50 mM Tris-HCl pH 7.5, 5 mM EDTA, 0.5% IGEPAL CA-630, 1.0% Triton X-100) on ice. Pellet was resuspended with IP buffer by pipetting and washed by centrifugation (12,000 g for 1 min at 4°C). To shear the chromatin, 1 mL of the washed and resuspended pellet was sonicated on ice. After centrifugation (12,000 g for 10 min at 4°C), supernatants were used for immunoprecipitation. Primary antibody (1 µg) was added to 1 ml of supernatant, and the samples were incubated for 12 hrs at 4°C on a rotating platform. IgG was used as a control antibody. After incubation, mixture of 20 µl of IP buffer and 20 µl of Protein G Agarose (Pierce) was added to the sample, and incubated for 45 min at 4°C on a rotating platform. After incubation, samples were washed five times by centrifugation (2,000 g for 3 min at 4°C), and the supernatants were removed. 100 µl of 10% Chelex 100 was added to the washed beads for DNA isolation, and the samples were boiled for 10 min at 90°C. After centrifugation (12,000 g for 1 min at 4°C), 80 µl of supernatant was transferred to new tube, and 120 µl of DDW was added to beads. After centrifugation (12,000 g for 1 min at 4°C), 120 µl of supernatant was collected and added to the previou**s** supernatant. Isolated DNA was used for PCR reaction. The primers were designed in the promoter region using Primer Quest Design tool from Integrated DNA Technologies, Inc. *Gapdh* was used as an control to validate this analysis. Used sequence is like below:

*Ache*, 5′ CCTTCTGCGTTCCACTATGT 3′ (forward)

5′ GGACACACTGACAGCTCTAATC 3′ (reverse)

*Gapdh*, 5′ TCGTCCGTCCTCTCTACTTT 3′ (forward)

5′ AGCTTTCTGGGCCTTCATAC 3′ (reverse)

#### Immunohistochemistry

Animals were perfused with ice-cold 0.09% normal saline (pH 7.4) for 20 min, followed by 4% paraformaldehyde. Coronal sections, measuring 40 µM in thickness, were cut through the prefrontal cortex to prepare serial sections using a freezing cryostat (Leica). The sections were collected in 24-well culture plates (Falcon, Becton Dickinson Labware S.A., Le Pont-de-Claix, France) containing 1 ml of tissue stock solution (glycerol and ethylene glycol in 0.2 M phosphate buffer) in each well. Sections were immersed in blocking buffer (10% Horse serum and 0.3% Triton X-100 in PBS) for 1 h at room temperature. The brain sections were incubated overnight at 4°C with primary antibody against AChE (Millipore, 1∶500), and rinsed 3 times with washing buffer (1.5% HS and 0.1% Triton X-100 in PBS) for 10 min. Secondary antibody conjugated with Alexa488 was diluted in blocking buffer and incubated for 2 h at room temperature. After 3 rinses with washing buffer, stained tissues were incubated with To-Pro3 (1∶1000) for nucleus staining, and were attached to coated slide glass and mounted in ProLong solution (Invitrogen, USA), before they were viewed using a confocal microscope (ZEN2009, Carl Zeiss).

#### Animals for *in vivo* and behavioral study

Pregnant Sprague-Dawley rats and ICR mice were purchased from OrientBio (Gyeonggi-do, Korea) and were maintained on a standard 12 hrs light-dark cycle, at ambient temperature (22±2°C) and humidity (55±5%) with free access to chow pellets and water. Prenatal exposure of VPA or PBS was performed as previously reported [29]. Rats and mice were injected with PBS or VPA subcutaneously at 400 mg/kg dosage on E12 and 300 mg/kg subcutaneously on E10, respectively. Total four mothers and their littermates were used for each group. Behavioral studies were performed from 4:00 PM to 8:00 PM. Before the behavior study, animals were habituated in the study place at least 1 hour.

Animal treatments including anesthesia, euthanasia and administration were carried out in accordance with the Principle of Laboratory Animal Care (NIH publication No. 85-23, revised 1985) and were approved by Animal Care and Use Committee of Konkuk University, Korea (KU13156).

### Subchronic drug treatment and behavioral studies

#### Drug treatment

Donepezil hydrochloride monohydrate (0.3 mg/kg of body weight) or saline was administered via intraperitoneal injection from postnatal day 14 to 40 (P14 to P40), once daily. Bodyweight was measured twice a week or before the behavior test. Dose was selected from the literature and preliminary test [36], [37]. In the preliminary test, 3 mg/kg and 5 mg/kg of the drug were injected to mice (P14) but soon after treatment some mice showed tremor and salivation (data not shown). Due to these side effects, we chose 0.3 mg/kg for this study. Moreover, chronic administration should be considered considering its regimen in clinical setting. The drug was freshly dissolved in saline (NaCl 0.9%) every administration schedule and given 30 min before starting the behavior test. During the experiment, no abnormal symptoms or toxic effects were observed in both drug and saline treated groups.

**Figure 1:**
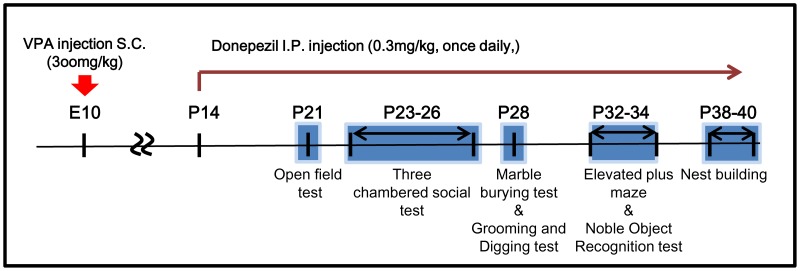
Drug treatment and behavioral study scheme with VPA mice. VPA was s.c. injected at embryonic day 10 (E10) to pregnant mice. Donepezil was i.p. injected once daily from postnatal day 14 (P14) until the end of the study. Behavioral studies were performed from P21. Grooming and digging test and novel object recognition tests were performed at 4 weeks of age with different sets of animals.

The 7 different types of behavioral analyses were performed consecutively until the animal reach P40 (Figure 1A). During the entire experimental periods, no body weigh differences were noted among experimental groups (Figure S3).

#### Open field locomotor test

Exploratory activity in a novel environment was assessed in an open field box (40×40×30 cm). Mice were introduced into the center area of the arena and given 5 min habituation before recording the actual behaviors. The total distance moved in the whole arena and the velocity were measured for 20 min using CCD camera-assisted motion tracking apparatus and software (EthoVision 3.1, Noldus Information Technology, the Netherlands). Ten mice in each group were used for this analysis (N = 10).

#### Three chamber social interaction test

The social interaction test was performed as previously reported [29]. The task consists of three sessions. In the first session, subject was introduced in the middle compartment and then habituated for 5 min. After habituation, a stranger animal (same age and no previous contact with the subject) of the same strain was introduced inside the wire cage of the left or right compartment (stranger zone 1) randomly while the other wire cage was empty (empty zone) for the 10 min sociability test. Time spent in the stranger zone 1 and around the cage was measured versus time spent in the empty zone. Social preference test was conducted for another 10 min directly after the termination of the sociability test. Another stranger animal was introduced in the wire cage of the opposite compartment (stranger zone 2) and same parameters were measured as with the previous session to find a preference of the subject animals to the novel over the familiar animal in the wire cage. The trace of movements during the experiment was automatically recorded using EthoVision software. Sociability (SI) and social preference indices (SPI) were calculated with the following formulas:
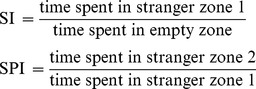



Ten mice in each group were used for this analysis (N = 10).

#### Elevated plus maze

Elevated plus-maze test was conducted according to the previously reported procedures [38]. The maze is composed of two open arms (30×5 cm), two closed arms (30×5×15 cm) and a central (5×5 cm) area. Mice were placed in the central area and allowed to move freely in the maze. Mice movement and time spent in the arms were automatically recorded using EthoVision software. From 8 to 11 animals were used for this analysis (N = 9 for Con and VPA, N = 8 for DPZ, N = 11 for V+D).

#### Marble burying test

The test was performed as previously reported with slight modifications [39]. Briefly, cages were filled with clean corncob bedding (1/8 inch, Anderson lab bedding, U.S.) with 3 cm height and the mouse was individually added for habituation. After 10 min, mouse was removed and 20 glass marbles (15 mm diameter) were carefully overlaid with equidistantly in a 4×5 arrangement. Each mouse was returned to its designated test cage and allowed to explore for 20 min. The number of marbles buried (>50% marble covered by the bedding) was recorded. Twelve mice in each group were used for this analysis (N = 12).

#### Self-grooming and digging test

The test was performed as previously reported with slight modifications [40], [41]. Before the experiment, each mouse was placed in the polycarbonate cage (20×26×13 cm) with clean corncob bedding (1/8 inch, Anderson lab bedding, U.S.) and habituated for 10 minutes. Accumulative time of grooming and digging behavior was measured simultaneously at a distance of 2 m from the cage for 10 minutes. Twelve mice in each group were used for this analysis (N = 12).

#### Novel object recognition test

The test was performed as previously reported [42]. Briefly, mice were placed in the empty polycarbonate cage (arena, 40×30×20 cm) and allowed to freely explore the cage for 10 minutes to habituate the experimental environment. The next day, identical two cylindrical plastic tubes (sample objects) were placed in the opposite corner. The subject was introduced into the arena carefully and given a period of 10 minutes to familiarize the sample objects. One hour later, one of the tubes was switched into a cubical novel object. And then the subject was re-introduced into the arena and recording the behavior for 5 minutes. Two observer evaluated their behavior by measuring the accumulative time interacting with each objects directly. The result was calculated as a discrimination ratio (novel object interaction/total interaction with both objects). Total 12∼13 mice in each group were used for this analysis (N = 13 for Con, VPA, and DPZ, N = 12 for V+D).

#### Nest building test

The subjects were individually housed in the polycarbonate cage (20×26×13 cm) 3 hrs before the experiment. In each cage, a nestlet (5×5 cm, Ancare, NY, U.S.) was added at 17:00 o'clock and the results were assessed by the trained experimenter with blind method in the next morning. Scoring was performed as previously described [43]. Eight mice in each group were used for this analysis (N = 8).

#### Acetylcholinesterease activity assay

AChE activity was measured at P35 using prefrontal cortex extracts after subchronic treatment of donepezil (N = 6). Briefly, mice were killed and prefrontal cortices were collected. Prefrontal cortices were homogenized with cold PBS on the ice. After centrifugation, supernatant was collected for the assay. To measure the AChE activity, we used Amplex Red ACh/AChE assay kit (Molecular probes, A12217) according to manufacturer's instructions. The measurement was performed using microplate reader (Spectramax Gemini EM, Molecular Devices) at excitation wavelength of 530 nm and emission wavelength of 590 nm. Background fluorescence was corrected by subtracting values from the negative control.

#### Statistical analysis

Data were expressed as mean ± standard error of mean (S.E.M) and analyzed for statistical significance using one-way analysis of variance (ANOVA) followed by Newman-Keuls test as a post-hoc test. Two-way ANOVA was used to identify the effect of VPA exposure or drug treatment, and interaction between the two factors. If significant effects were found in any one of the factors, post-hoc comparisons were conducted using Bonferroni's post-tests. Differences were considered statistically significant when the p value was less than 0.05 (*p*<0.05). All statistical analyses were conducted using GraphPad Prism (version 5) software.

## Supplementary Materials and Methods

### Single drug treatment and behavioral studies (Figure S1)

Two dose of donepezil hydrochloride monohydrate (0.3 mg/kg and 1 mg/kg) or saline was administered to mice based on bodyweight at 30 minutes just before the behavior experiments. Open field locomotor test and three chamber social behavior test was performed as described above. Open field locomotor test was performed at P23 (N = 10) and three chambered social behavior test at P30 to 32 (N = 11 for Con, N = 10 for VPA, N = 8 for V+D 0.3 mpk (VPA+donepezil, 0.3 mpk), N = 10 for V+D 1 mpk (VPA+donepezil, 1 mpk)).

## Results

### Prenatal exposure of VPA induced up-regulation of AChE in the prefrontal cortex of rodent offspring

**Figure 2:**
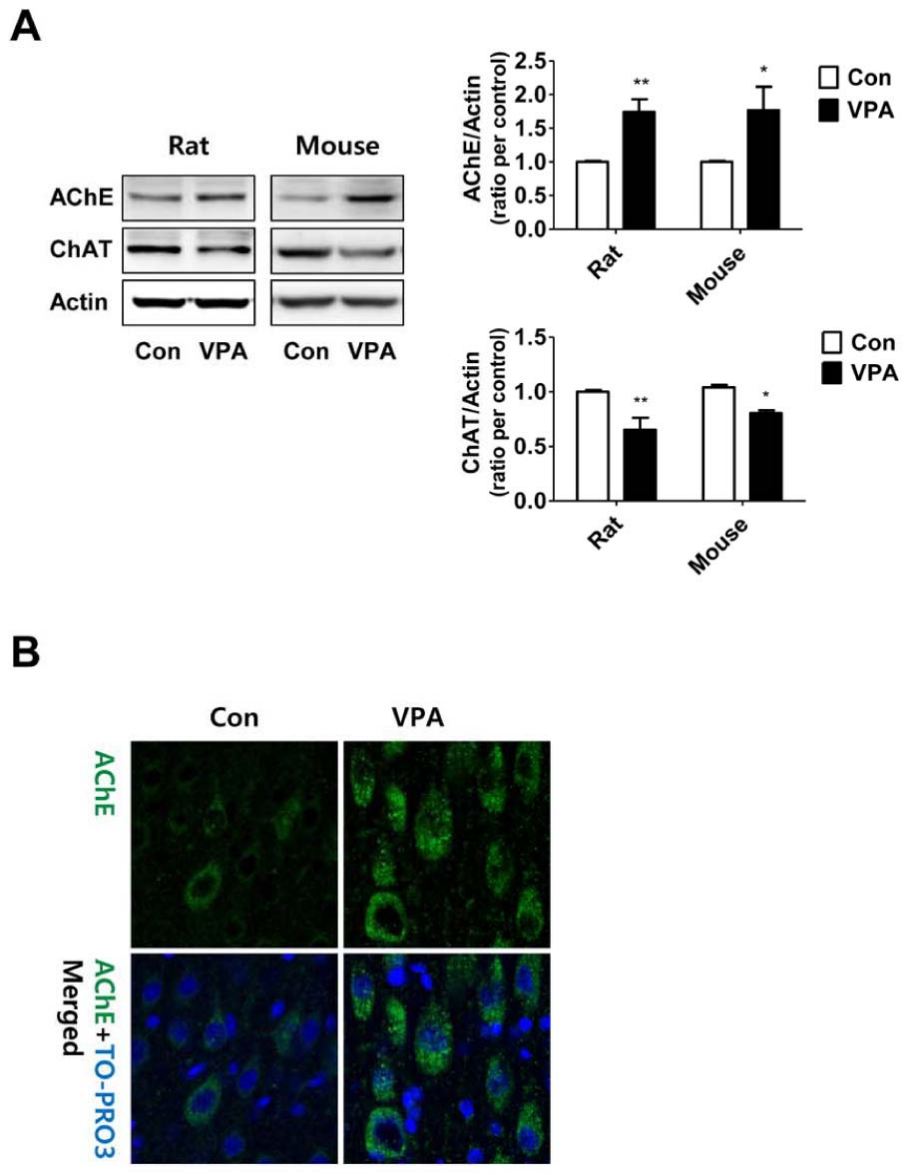
Dysregulation of cholinergic enzyme in the prefrontal cortex region of VPA induced animal model of ASD. Expression level of acetylcholinesterase and choline acetyltransferase was measured using Western blot (A), and acetylcholinesterase was also measured using immunohistochemistry (B) in the prefrontal cortex at post natal day 28. (A) Prenatally VPA-exposed SD rats (400 mg/kg) and ICR mice (300 mg/kg) showed increased acetylcholinesterase (AChE) but decreased choline acetyltransferase (ChAT); left panel rat (N = 6), right panel mouse (N = 4). Data are expressed as the mean ± S.E.M. *, **, *p*<0.05 and *p*<0.01 vs. control. (B) Immunohistochemical staining against acetylcholinesterase (green) in prefrontal cortex region from SD rat exposed to VPA at prenatal period. Nucleus was counter-stained with TO-PRO3 (blue).

Because the prefrontal region is known as an important area for social recognition and behavior [44], we investigated expression level of ChAT and AChE, a rate limiting enzyme for the synthesis and primary metabolic enzyme of ACh, respectively, using Western blot in the prefrontal cortex region of rat and mouse offspring at week 4 (Figure 2A). In rat prefrontal cortex, AChE level in the VPA treated group was significantly higher than control group (1.74±0.19 fold vs control, p<0.01). In contrast, ChAT level was slightly but significantly decreased in the VPA treated group (0.73±0.12 fold vs control, p<0.05). Similar results were observed in the prefrontal cortex of mouse offspring (Figure.2A). The increased AChE expression was also identified using immunohistochemistry (Figure 2B).

### Histone deacetylase inhibitors induced up-regulation of AChE in cultured rat cortical neural progenitor cells

**Figure 3:**
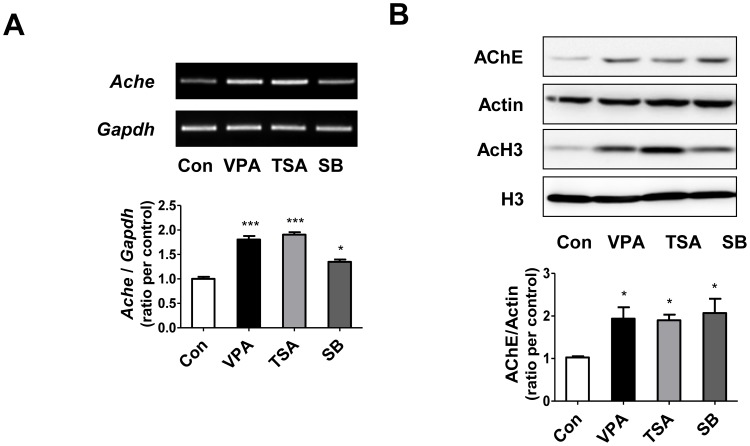
Histone deacetylase inhibitors increased expression of AChE in cortical NPCs from SD rat. Cortical neural progenitor cells from SD rat were treated with HDACIs: VPA (0.5 mM), TSA:Trichostatin A (0.1 µM), and SB:Sodium butyrate (0.1 mM). After 24 hours, mRNA and protein expressions were analyzed using RT-PCR (A) and Western blot (B). (A) *Ache* mRNA expression level was increased by both VPA and other HDACIs (N = 3). (B) Protein expression of AChE was also increased by VPA and other HDACIs treatment (N = 4). Data are expressed as the mean ± S.E.M. *, ***, *p*<0.05 and *p*<0.001 vs. control (*Ache*:Acetylcholinesterase mRNA, *Gapdh*:GAPDH mRNA, AChE:Acetylcholinesterase, AcH3:acetylated Histone H3, H3:Histone H3, VPA:valproic acid, TSA:Trichostatin A, SB:Sodium butyrate).

To elucidate the underlying mechanism responsible for the increased AChE expression, we treated VPA (0.5 mM, a well-known HDACI) and other HDACIs, trichostatin A (0.2 µM) and sodium butyrate (0.1 mM), to cultured cortical neural progenitor cells (NPCs) obtained from SD rat embryos (E14). After drugs treatment, NPCs were incubated for 24 hrs in differentiating condition (without growth factors) and expression level of AChE was measured by RT-PCR (Figure 3A) and Western blot (Figure 3B). *Ache* gene expression level (Figure 3A) was increased by HDACIs treatment (fold increase, control vs VPA group  = 1.81±0.12 fold, p<0.001, TSA = 1.91±0.09 fold, p<0.001, SB = 1.35±0.08 fold, p<0.05). AChE protein level (Figure 3B) was also increased by HDACIs (fold increase, control vs VPA = 1.94±0.53, p<0.05, TSA = 1.90±0.27, p<0.05, SB = 2.07±0.67, p<0.05). We also confirmed that global acetylation of histone H3 was increased by treatment of HDACis (N = 4).

### Increased acetylation of histone H3 bound to AChE gene promoter region in VPA autistic animal model

**Figure 4:**
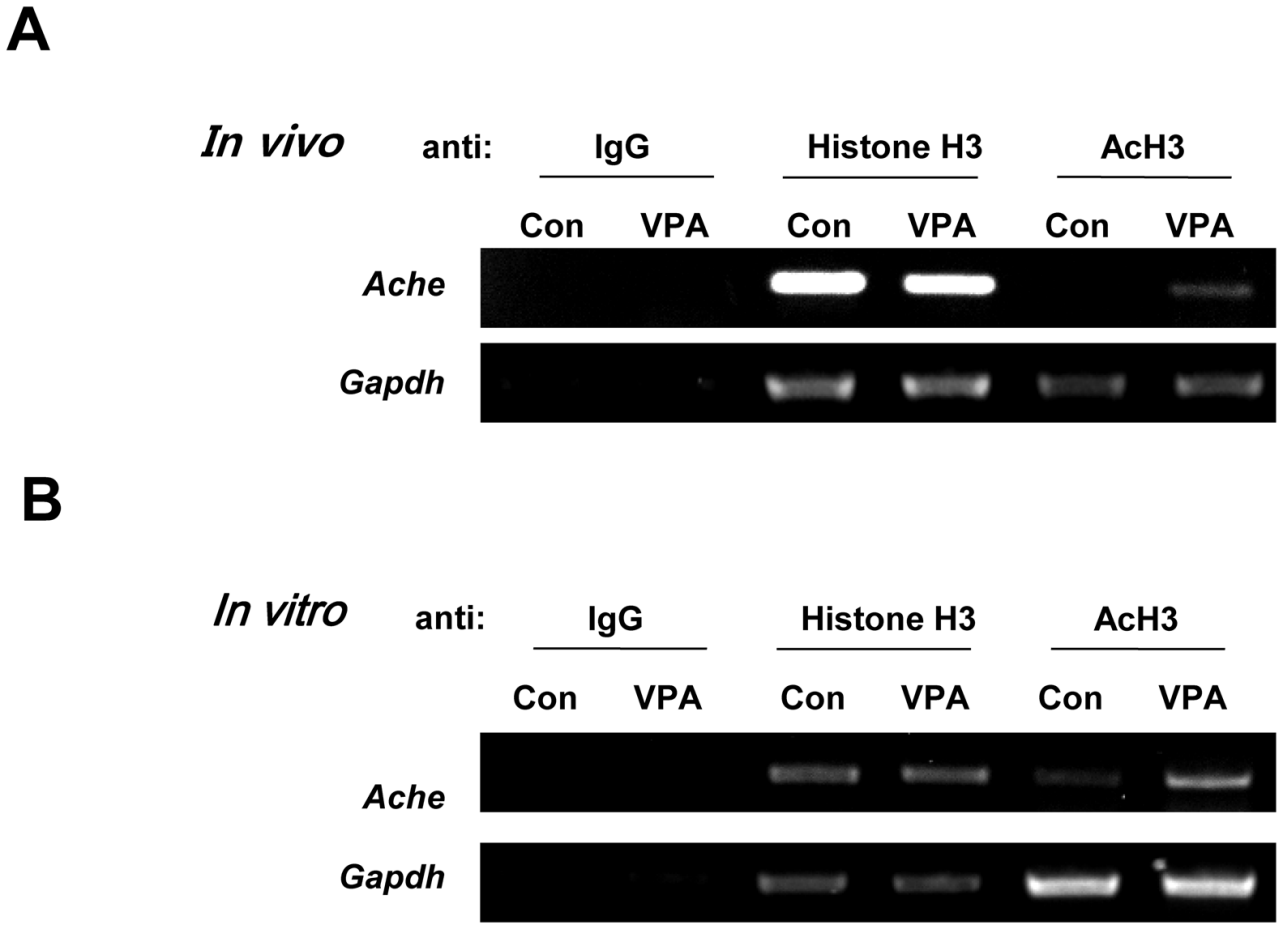
Acetylation of histone H3 bound to promoter region of *Ache* was increased by VPA treatment. To confirm the effect of HDACIs on the upregulation of AChE, ChIP was performed using prefrontal cortex from VPA rat and VPA treated cortical NPCs from SD rat. (A) ChIP results using prefrontal cortex of rat prenatally exposed to VPA or PBS (con) (B) ChIP results from cortical neural progenitor cells of rat. *Gapdh* was used as a positive control for this analysis. (AcH:acetylated Histone H3, Ache:acetylcholinesterase).

VPA is a well known HDACI and acetylated histone was known to play a role in the activation of gene transcription [45]. Therefore, we hypothesized that increased AChE level might be mediated by hyperacetylated histone H3 level in *Ache* promoter region [46]. To prove this, we investigated interaction between acetyl histone H3 and the promoter region of *Ache* gene using ChIP with prefrontal cortex region obtained from VPA rat (Figure 4A) as well as rat NPCs treated with VPA (Figure 4B). *Gapdh* DNA was used as a positive control in both experiments. We found that acetyl histone H3 binding to *Ache* gene promoter region was more pronounced in the prefrontal cortex region of VPA animal model and cortical NPCs treated with VPA. These results suggest that prenatal exposure of VPA caused hyperacetylation in histone H3, which activated *Ache* gene expression by epigenetic mechanism.

### Subchronic treatment of donepezil improved social behavioral deficits in the VPA animal model mice

**Figure 5:**
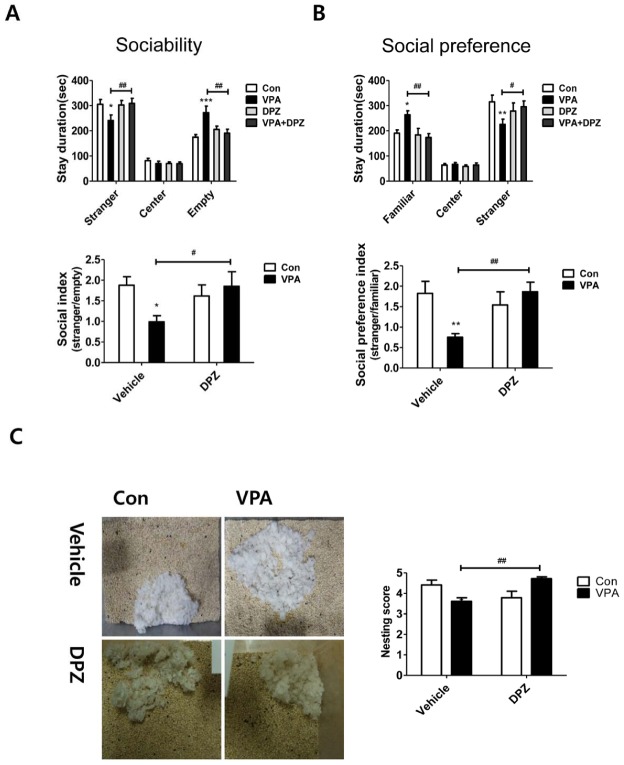
Subchronic treatment of donepezil improved social behavior in VPA exposed mice. In three chamber assay, sociability (A) and social preference (B) were measured. Upper panel showed stay duration (sec) in each compartment and lower panel showed social index and social preference index (N = 10). (C) Nest building test. The built nests during 16 hours period were scored by trained experimenters who were blind to the experimental conditions (1, poor; 5, good), N = 10. Data are expressed as the mean ± S.E.M. *, **, ***, *p*<0.05, *p*<0.01, *p*<0.001 vs. control in same compartment. #, ##, ###, *p*<0.05, *p*<0.01, *p*<0.001 vs. VPA exposed mice in same compartment.

Since increased AChE level may adversely affects the level of ACh in brain, we hypothesized inhibition of AChE by using its inhibitor, donepezil, may improve autistic symptoms in the VPA-induced autistic animal model. We treated donepezil subchronically with once daily regimen from P14 in VPA mice. About one week later (P23), social behavioral abnormality was investigated. To check the social deficits, two types of social behavior tests were performed using three-chamber assay (Figure 5A and B). In the first session, we assessed sociability by measuring staying time in the compartment with a stranger mouse placed in small wire cage or empty wire cage. In the second session, a new mouse was added in the previous empty compartment and social preference between familiar mouse and novel mouse was assessed by measuring staying time between familiar and stranger mice. Based on the data, we calculated sociability index and social preference index as previously described [29]. In the sociability test (Figure 5A), VPA mice stayed more time in the empty space than mice in other groups and their staying time in the compartment with a conspecific mouse was significantly lower than control mice suggesting the deficits in sociability. Interestingly, VPA group treated with donepezil stayed more time in the compartment with a conspecific mouse. The results were also calculated as sociability index, which showed improved social interaction by the donepezil treatment (F(1,36) = 4.80, p<0.05) in VPA group. In social preference test (Figure 5B), VPA group stayed significantly more time with familiar mice and less time with stranger mice than control group. VPA group treated with donepezil showed similar stay duration in those compartments as compared with control. The social preference index showed the improvement of social preference in VPA group treated with donepezil (F(1,36) = 7.71, p<0.01).

We also evaluated the social behavior by measuring approaching time (sniffing time) to either wire cage with mouse or empty wire cage (Figure S2A). VPA mice showed significantly lower social approach index and social preference approach index. Donepezil treated mice approached more time near the stranger mouse in both sociability test and social preference test. Although approach index of donepezil treated VPA mice did not show statistical significance compared with VPA group, they showed clear tendency of improvement in their social behavior.

To confirm the improved social behavioral effects of donepezil in VPA group, we assessed nest building score according to previous reports (Figure 5C) [47], [48]. We placed nestlet in the middle of cage at 17:00 o'clock and scored the built nest using blind method at 9:00 o'clock in the next morning. Nest score of VPA group was lower than control group (Con = 4.42±0.66, VPA = 3.60±0.51, p<0.05). Donepezil treatment in VPA group showed a significantly improved nest score (VPA = 3.60±0.51, VPA+DPZ = 4.71±0.26, F(1,27) = 16.30, p<0.001).

### Subchronic treatment of donepezil improved repetitive behavior in the VPA animal model

**Figure 6:**
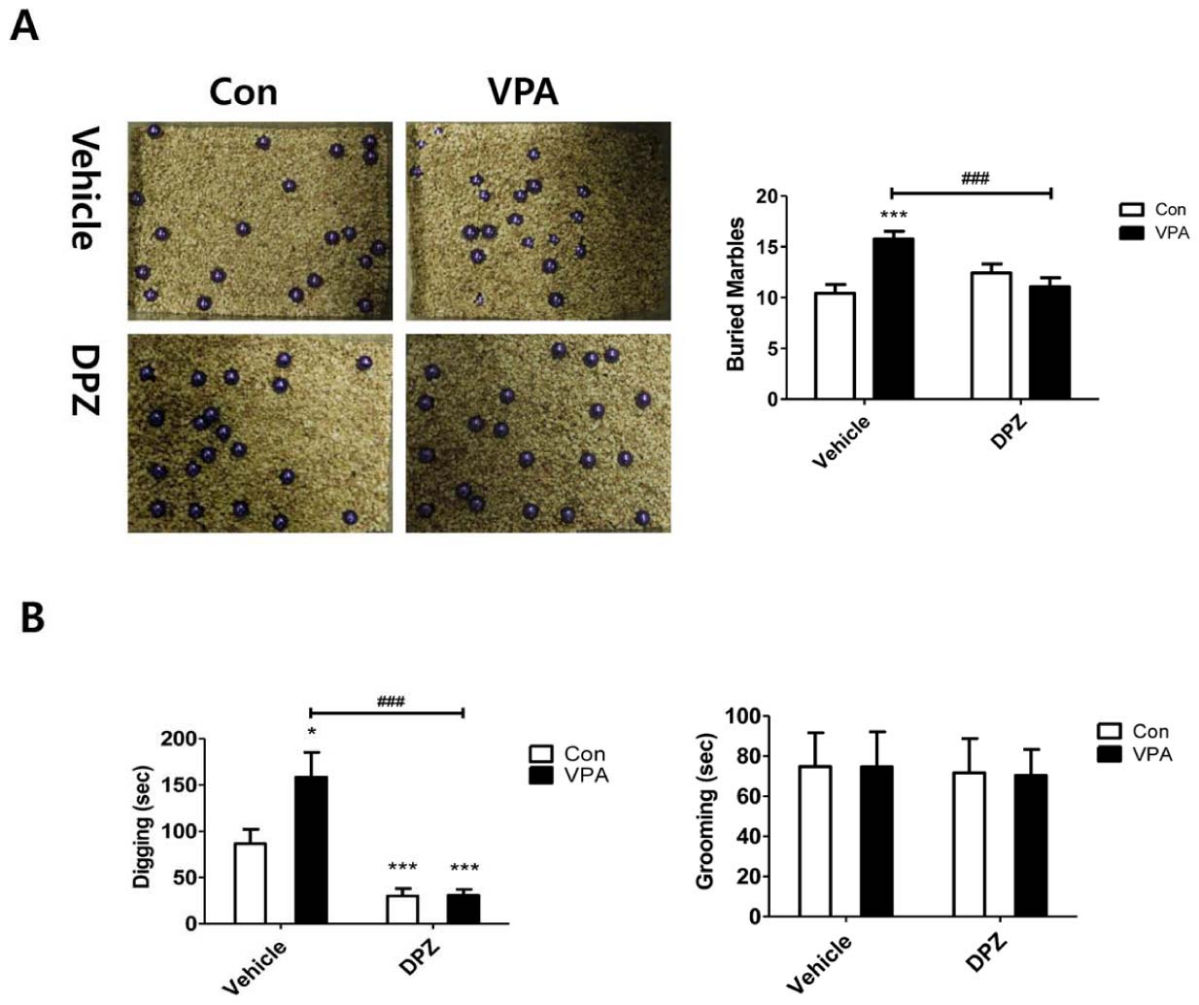
Subchronic treatment of donepezil improved repetitive behaviors in VPA exposed mice. (A) Marble burying test. Twenty marbles were equidistantly arranged (5×4) on the bedding surface. After 20 min, the number of buried marbles (>50% of its surface) was measured (N = 12). (B) Digging and grooming behavior tests were performed by simultaneously observing the home cage behavior of mice. Each mouse was placed individually in a mouse cage with 1 cm layered bedding. After 10 min habituation, accumulative time of digging and grooming behavior was measured for another 10 min (N = 12). Data are expressed as the mean ± S.E.M. *, ***, *p*<0.05, *p*<0.001: control mice vs. VPA mice. ###, *p*<0.001: VPA mice vs. donepezil treated VPA mice.

As previously reported, to investigate repetitive behavioral phenotype, we performed marble burying test (Figure 6A) [39]. VPA mice buried more marbles than control group but donepezil treated VPA group buried marbles at the same level as compared with control group (F(1,44) = 15.08, p<0.001).

To confirm the result, we also performed digging and grooming behavior test at P28 using another test sets of animals (Figure 6B). Cumulative digging and grooming time was measured simultaneously during the experiment. VPA mice showed more digging behavior than the control group did (p<0.05). But the digging behavior was reduced in donepezil treatment groups (F(1,44) = 31.72, p<0.0001). On the other hand, no significant differences were observed in the grooming behavior.

### Subchronic treatment of donepezil improved hyperactive behavior in the VPA animal model

**Figure 7:**
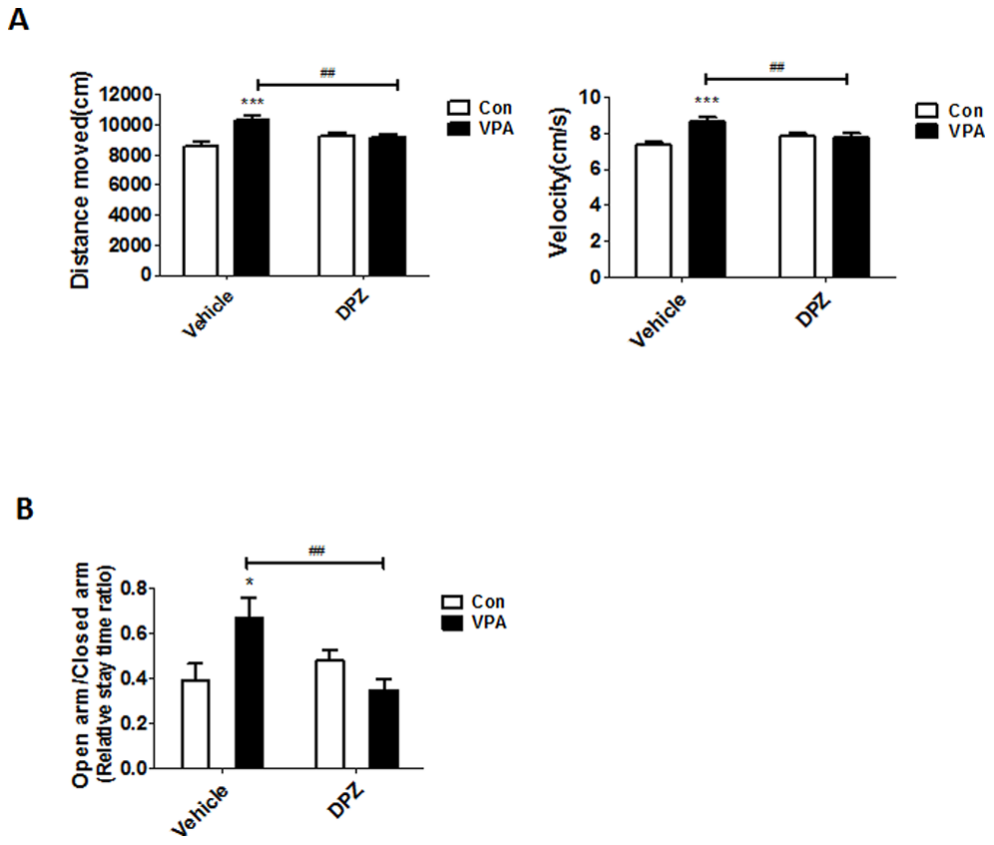
Subchronic treatment of donepezil improved hyperactive and abnormal anxiety behavior in VPA exposed mice. (A) Open field test. Locomotor activity was measured for 20 min using CCD camera-assisted motion tracking apparatus and software (EthoVision 3.1, Noldus information Technology, the Netherlands). Left panel showed total distance moved and right panel showed their velocity (N = 12). (B) Elevated plus maze. Data expressed as a ratio of staying time in open arms divided by staying time in closed arms (N = 8 for Con and VPA, DPZ:8, N = 11 for V+D). Data are expressed as the mean ± S.E.M. *, ***, *p*<0.05, *p*<0.001: control mice vs. VPA mice. ##,###, *p*<0.01, *p*<0.001: VPA mice vs. donepezil treated VPA mice.

In the open field test, VPA mice displayed significantly greater locomotor activity but the increased locomotor activity was significantly reduced by donepezil treatment (F(1,44) = 16.20, P<0.001). Moreover, the velocity of movement in VPA group was also higher than control mice, which was significantly reduced in donepezil treated group (F(1,44) = 12.33, p<0.01) (Figure 7A).

To identify their anxiety-related behavior, we performed the elevated plus maze test. Interestingly, VPA mice stayed more time in the open arm than control mice suggesting less-anxious behavior but donepezil treatment restored the abnormal anxiety level in VPA group to control level (F(1,33) = 9.14, p<0.01)(Figure 7B).

### Subchronic treatment of donepezil improved cognitive rigidity in the VPA animal model mice

**Figure 8:**
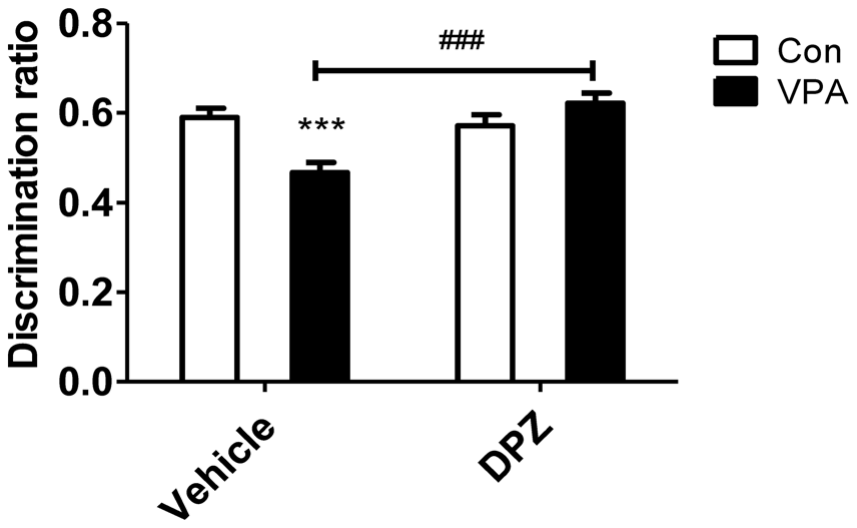
Subchronic treatment of donepezil improved impaired recognition in VPA exposed mice. Novel object test was performed as described in methods. Data are expressed as a discrimination ratio (novel object interaction/total interaction with both objects, N = 12∼13, the mean ± S.E.M.) ***, *p*<0.001: control mice vs. VPA mice. ###, *p*<0.001: VPA mice vs. donepezil treated VPA mice.

In the recent report, subchronic donepezil treatment alleviated the cognitive rigidity in the BTBR mice, a well-known autistic mice model [49]. So, we wanted to identify the effects of donepezil using novel object recognition test in VPA animal model (Figure 8). Interestingly, VPA mice showed significantly reduced cognitive flexibility but the deficits were rescued by subchronic donepezil treatment to control level (F(1,47) = 14.27, p<0.001).

### Subchronic treatment of donepezil corrected increased AChE activity in the VPA animal model mice

**Figure 9:**
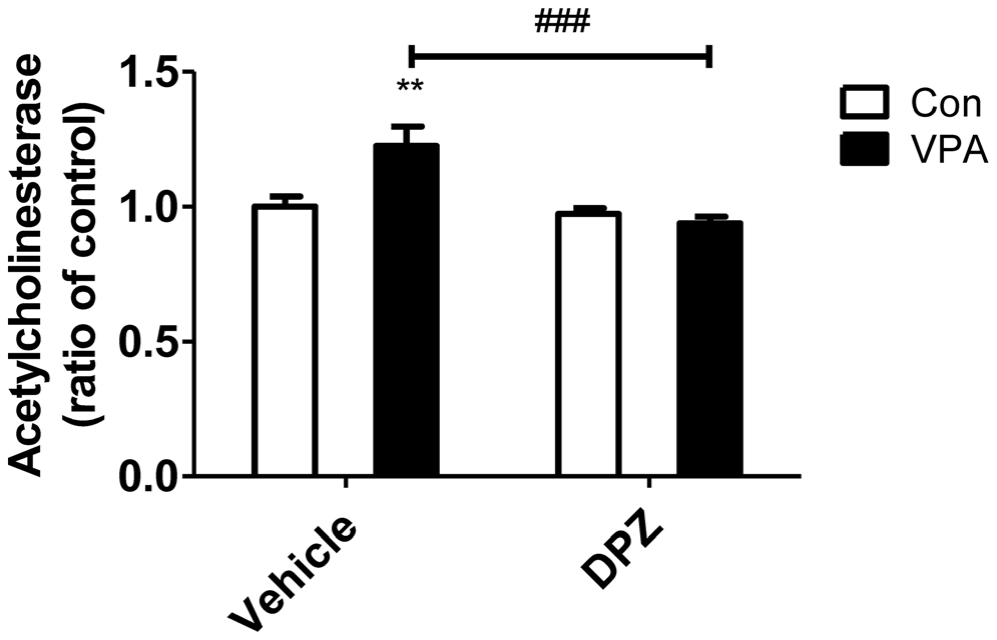
Subchronic treatment of donepezil corrected increased AChE activity in the VPA exposed mice model. AChE activity was measured in the prefrontal cortex at P35 (N = 6). Data are expressed as the mean ± S.E.M. **, *p*<0.01: control mice vs. VPA mice. ###, *p*<0.001: VPA mice vs. donepezil treated VPA mice.

To confirm the pharmacological effect of donepezil, we measured the AChE activity from the prefrontal cortex after subchronic treatment of donepezil at P35 (Figure 9). In VPA group, increased AChE activity was observed but treatment of donepezil reduced the increased AChE activity in the VPA mice to the control level (F(1,20) = 8.69, p<0.001).

## Discussion

Therapeutics for all core-symptoms of ASD such as social deficit, language impairments, and repetitive or restricted behaviors are not yet fully available, thus giving a formidable task to many researchers studying ASD. Since there are a multitude of animal models for ASD due to its heterogeneity, to screen out therapeutic potentials of candidates with specific animal model might have limited applicability [50]. However, those studies have provided us understanding of the pathophysiological conditions or relationship between therapeutics and behavioral improvements in ASD. In this study, we found that prenatal exposure of VPA, an animal model of ASD, caused up-regulation of AChE. The behavioral symptoms such as social deficit, repetitive, hyperactive and anxiety-like behaviors in VPA group were all improved by donepezil, a clinically available pharmacological treatment to correct abnormal cholinergic transmission.

In this study, we used prenatal VPA exposure animal model of ASD, which has been widely used by various researchers including us. It was widely reported that VPA exposure during the neural tube closure period showed high incidence of autistic behavioral symptoms such as reduced social behavior, repetitive behavior, hyperactive behavior, and impaired recognition in rodent offspring [29], [30], [51]. These effects of VPA also have been known in humans as fetal valproate syndrome. Children exposed to VPA *in utero* showed congenital malformations, tube defects, brain anomalies, eye anomalies, developmental delays and autistic behaviors [52], [53], [54]. For these reasons, VPA has been regarded as a potent environmental risk factor for ASD and is widely used in an ASD animal model.

In our study, we found that prenatal exposure of VPA upregulated AChE expression in the neural progenitor cells and the aberrantly upregulated enzyme expression was persistent even in the postnatal period. This result seems to be mediated by epigenetic regulation of VPA. VPA is a HDACI [55] and hyperacetylated histone correlates with activation of transcription [56]. Hyperacetylated histone-dependent sustained epigenetic dysregulation could be found in physiological processes or neuropsychiatric disorder conditions. Similar to our results, Sailaja et al. reported that HDACIs and stress induced activation of AChE expression for a long period via sustained hyperacetylated histone modification [57]. We speculate that HDAC inhibition or strong maternal stress may underlie as an environmental etiological factor of ASD, which might result in dysregulation of cholinergic system such as activated AChE expression via epigenetic regulation. Although further study might be needed to unequivocally prove cholinergic deficits in our model, we primarily suggest that dysregulated metabolic and synthetic enzyme could result in dysregulated cholinergic neurotransmission, which might be similar to the condition found in Alzheimer's disease.

In this study, we tried donepezil to identify its therapeutic potential in VPA exposed animals. Considering the age of ASD diagnosis and need for therapeutic intervention at an early age, we administrated donepezil subchronically to VPA mice from P14. We found that subchronic treatment of donepezil improved performance of VPA mice in various tests related to social, repetitive, hyperactive and recognition behaviors. Although behavioral analysis have limitations to translate the underlying neurological mechanisms, the behavior tests that we performed have been well-known as battery tests to investigate autistic behaviors in animal models (reviewed in [47]).

The subchronic treatment of donepezil improved social behavior and social memory represented by the three chamber social tests and nest building test. Previous reports suggested that impaired cholinergic condition causes cognitive problems and may also cause social problems, which were rescued by donepezil treatment [37], [49]. Consistent to these reports, in our study, VPA mice showed impaired recognition behavior not only in the novel object recognition test but also in the social preference test. These impaired behaviors were however reversed by subchronic donepezil treatment and we further confirmed that the overly increased AChE in VPA exposure was also reduced by donepezil treatment. From these results, we might assume that modulation of cholinergic transmission by subchronic treatment of donepezil may at least in part contribute to the observed therapeutic effects. However, this conclusion should be carefully verified in the future study since cholinergic deficits cannot be assumed automatically to be solely based on the abnormal expression levels of ChAT or AChE because the animal model with altered ChAT or AChE did not consistently show deficit in cholinergic neurotransmission. Thus, cholinergic transmission should be carefully examined by using more direct techniques such as high affinity choline uptake as well as microdialysis in future experiments. In addition, the effects of other cholinergic mimetics including AChEIs such as revastigmine and galantamine may provide valuable information on the role of cholinergic transmission in the modulation of ASD-like behaviors.

In the elevated plus maze study, VPA mice stayed more time in open arm than closed arm. Although elevated plus maze usually reflects the anxiety-related behavior, excessive preference to open arms may also represent impulsive behavior [58]. Reduced anxiety level to novel or risky environment could be induced from lower level of cognition possibly due to the decreased level of ACh. In previous studies, hypomorphic and null alleles of TSC2 mice, another model of ASD, showed excessive preference to open arms. Furthermore, they also noted that reduced anxiety levels in elevated plus maze has a connection with impulsive behavior [59]. Investigating cholinergic deficits in animal models showing reduced anxiety or increased impulsive behavior may present the connection between neurochemical conditions and behavioral symptoms in ASD.

Interestingly, cholinergic deficit is also found in other ASD animal model, the BTBR mice. This mouse displays ASD core symptom behaviors; impaired social behavior and ultrasonic vocalization with repetitive grooming behavior [40], [60], [61], [62]. In a recent report, lower acetylcholine level was observed in the prefrontal cortex region of mice which showed attention deficit and impulsive behavior [63]. The elevation of ACh level by subchronic treatment of donepezil improved social deficiency and cognitive rigidity in these mice [49]. Transgenic mouse study also supports the role of AChE on abnormal behaviors. Transgenic mice harboring overexpression of human AChE-S showed impaired social recognition behavior, increased locomotor activity in the novel open field space and increased staying time in open arms of elevated plus maze [64], [65]. These studies agree with our findings and support the possibility of cholinergic drug as a potential therapeutic target for ASD.

In our previous reports, we found that VPA-exposed animals showed increased glutamatergic neuronal differentiation and structures such as PSD95 and vGluT1 in the prefrontal cortex region [32]. Rinaldi et al. also found that NR2A and NR2B was upregulated in the neocortex region of VPA animals [66]. Consistent with these findings, we observed an improved social and hyperactive behaviors and lowered electric seizure sensitivity by memantine treatment in our previous study [32]. Since memantine is an NMDA blocker, memantine treatment would down regulate overly expressed glutamatergic signaling in VPA animal models. Interestingly, donepezil treatment in therapeutic concentration decreased NMDA currents in an *in vitro* study using cortical neuron [67]. In addition, donepezil also caused internalization of NMDA receptor from cellular surface via α7 nAChRs in primary cortical neurons [68]. Similarly, we also found that increased NR1 expression in VPA mice prefrontal cortex was downregulated to control level by donepezil treatment in Western blot analysis (data not shown). These evidences suggest that the excitatory/inhibitory imbalance theory of ASD could be another underlying therapeutic target of donepezil treatment. Whether the effects of donepezil observed in this study may also be applicable in a wider concept of E/I imbalance would be another interesting topic to study further in the future.

Although cholinergic deficit was also observed in other neuropsychiatric disorders, we focused on ASD using VPA animal model in this study. In this study, we investigated the therapeutic potential for ASD using cholinergic modulating drug, donepezil. Since ACh mediates various functions in neurotransmission and neuronal development in the brain, cholinergic deficit would cause disconnection of synaptic network and result in abnormal behavioral symptoms. Although more studies are required to unequivocally answer the therapeutic pathway in the dysregulated cholinergic transmission of ASD, our study suggests that modulation of cholinergic system can be a new therapeutic target to handle ASD core symptoms. The question whether those drugs used for neurodegenerative disorders like Alzheimer's disease could also be effective in neurodevelopmental disorders such as ASD, and what could be their possible mechanisms of action, should be answered in future studies.

## Supporting Information

Figure S1 **Single acute treatment of donepezil improved hyperactive behavior but not social behavior in VPA exposed mice.** (A) Open field test. Open field test was performed at P23 (N = 10). (B) Three chamber assay for the measurement of sociability and social preference. Three chamber assay were performed at P30∼P33 (Con: N = 11, VPA: N = 10, V+D 0.3 mg/kg: N = 8, V+D 1 mg/kg: N = 10). Data are expressed as the mean ± S.E.M. *, **, ***, *p*<0.05, *p*<0.01, *and p*<0.001: vs. control or control in the same compartment. #, *p*<0.05, vs. VPA exposed mice. (V:VPA, D:donepezil).
(TIF)

Figure S2 **Abnormal social behavior as determined by approaching (sniffing) time was improved by chronic donepezil treatment in VPA exposed mice (related to** **figure 4A and B** **).** Sociability and social preference were re-analyzed based on approaching (sniffing) time to the stranger mouse or the empty wire cage. In each trial, observer measured the time spent near the wire cages and the sniffing time. Data are expressed as the mean ± S.E.M. *, *p*<0.05, vs. control in same compartment. #, *p*<0.05, vs. VPA exposed mice.
(TIF)

Figure S3 **Body weight change trajectory in the donepezil subchronic treatment study.** There were no significant body weight changes among groups.
(TIF)
