# Supplementary figures and images for: Subchronic Treatment of Donepezil Rescues Impaired Social, Hyperactive, and Stereotypic Behavior in Valproic Acid-Induced Animal Model of Autism
Source: PLoS One. 2014 Aug 18;9(8):e104927. doi: 10.1371/journal.pone.0104927 (PMC4136791; doi:10.1371/journal.pone.0104927)

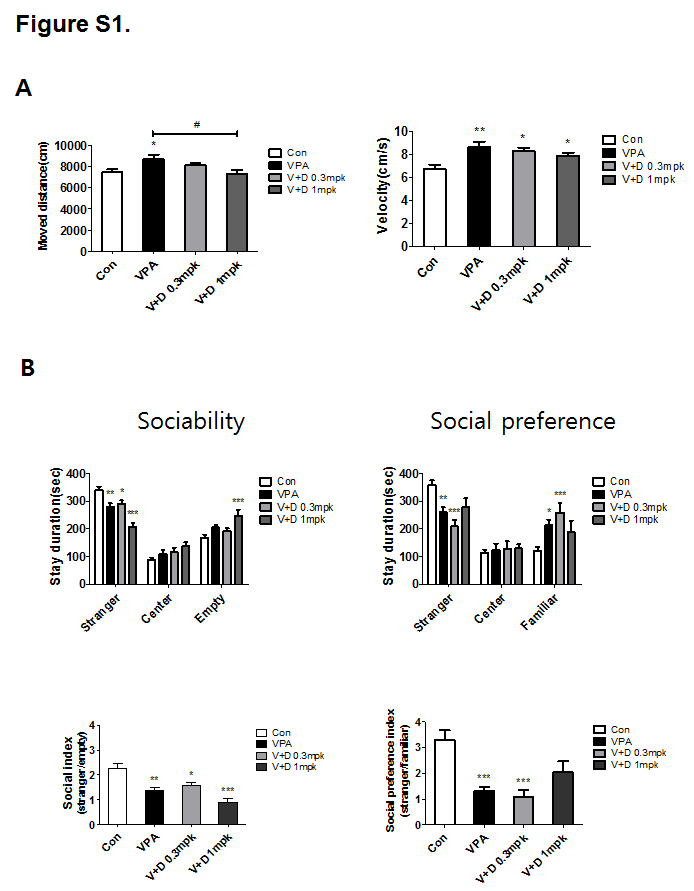

Supplement: Figure S1 — Single acute treatment of donepezil improved hyperactive behavior but not social behavior in VPA exposed mice. (A) Open field test. Open field test was performed at P23 (N = 10). (B) Three chamber assay for the measurement of sociability and social preference. Three chamber assay were performed at P30∼P33 (Con: N = 11, VPA: N = 10, V+D 0.3 mg/kg: N = 8, V+D 1 mg/kg: N = 10). Data are expressed as the mean ± S.E.M. *, **, ***, p<0.05, p<0.01, and p<0.001: vs. control or control in the same compartment. #, p<0.05, vs. VPA exposed mice. (V:VPA, D:donepezil). (TIF) [file pone.0104927.s001.tif]

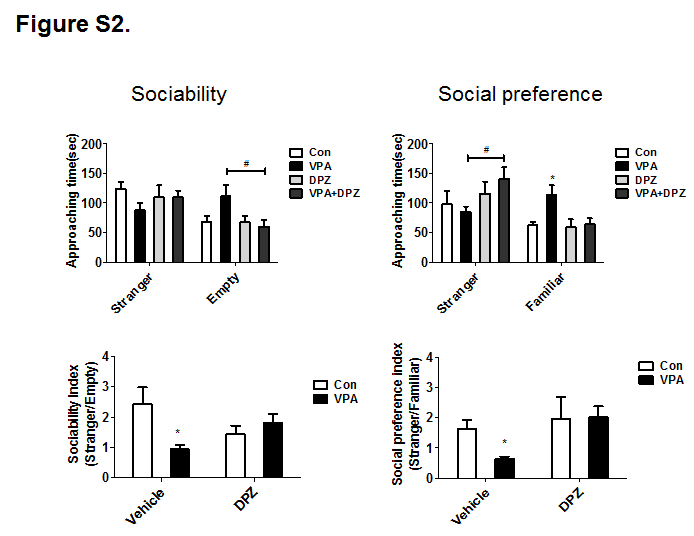

Supplement: Figure S2 — Abnormal social behavior as determined by approaching (sniffing) time was improved by chronic donepezil treatment in VPA exposed mice (related to figure 4A and B ). Sociability and social preference were re-analyzed based on approaching (sniffing) time to the stranger mouse or the empty wire cage. In each trial, observer measured the time spent near the wire cages and the sniffing time. Data are expressed as the mean ± S.E.M. *, p<0.05, vs. control in same compartment. #, p<0.05, vs. VPA exposed mice. (TIF) [file pone.0104927.s002.tif]

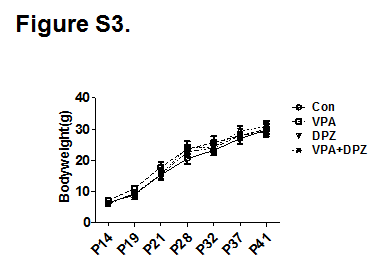

Supplement: Figure S3 — Body weight change trajectory in the donepezil subchronic treatment study. There were no significant body weight changes among groups. (TIF) [file pone.0104927.s003.tif]
